# In-hospital clinical outcomes in diffusion weighted imaging-negative stroke treated with intravenous thrombolysis

**DOI:** 10.1186/s12883-022-02878-w

**Published:** 2022-09-15

**Authors:** Guangshuo Li, Xueyan Feng, Chuanying Wang, Yahui Hao, Shang Wang, Yunyun Xiong, Xingquan Zhao

**Affiliations:** 1 Department of Neurology, Beijing Tiantan Hospital, Capital Medical University, No. 119 Nansihuanxilu, Fengtai District, Beijing, 100070 China; 2 China National Clinical Research Center for Neurological Diseases, Beijing, China; 3 Chinese Institute of Brain Research, Beijing, China

**Keywords:** Stroke, Thrombolysis, Tissue plasminogen activator, Diffusion magnetic resonance imaging

## Abstract

**Objective:**

We aimed to investigate whether negative diffusion weighted imaging (DWI) is related to the in-hospital clinical outcomes for ischemic stroke patients with intravenous tissues plasminogen activator (IV tPA).

**Methods:**

We retrospectively enrolled patients who received IV tPA therapy within 4.5 hours from symptoms onset. The classification of DWI-positive or negative was based on post-IV tPA MR scan. Demographic factors, stroke characteristics, imaging information, and the in-hospital clinical outcomes including early neurological improvement (ENI) and favourable functional outcome were collected. Multivariable logistic regression and sensitivity analyses were conducted to test whether negative DWI imaging was an independent predictor of the in-hospital clinical outcomes.

**Results:**

In the final study population, 437 patients treated with IV tPA were included and 12.36% of them had negative DWI imaging at the first MR scan post IV tPA. In the DWI-negative group, 51.9% (28/54) of the patients achieved ENI at 24 hours and 74.1% (40/54) of the patients achieved favourable clinical outcome at discharge. DWI-negative was not related to ENI (adjusted odds ratio 0.93, 95% confidence interval 0.17–4.91) or favourable clinical outcome (adjusted odds ratio 2.40, 95% confidence interval 0.48–11.95). Additional sensitivity analyses yielded similar results.

**Conclusion:**

DWI-negative is not associated with ENI or favourable functional outcome at discharge.

**Supplementary Information:**

The online version contains supplementary material available at 10.1186/s12883-022-02878-w.

## Introduction

Diffusion-weighted imaging (DWI) of magnetic resonance imaging (MRI) has enabled neurologists to diagnose ischemic stroke within minutes [1] with high sensitivity (over 80%) and specificity (over 95%) [2]. Since 2010, the American Academy of Neurology (AAN) has recommended DWI as ‘the most accurate diagnostic imaging method of ischemic stroke’ [3].

However, a meta-analysis [4] identified a minority (6.8%) of ischemic stroke patients with normal DWI imaging. In the first 24 hours from stroke onset, particularly in a stroke of the posterior circulation, nearly 20% of acute ischemic changes were missing on DWI imaging [5]. Intravenous thrombolysis with intravenous tissues plasminogen activator (IV tPA) shall be initiated rapidly after ischemic stroke attack [6] and treatment delay would weaken the effect of IV tPA [7]. MR scan, a more time-consuming imaging tool, is less generalized than CT scan in screening eligible patients for IV tPA. Hence, patients that could have a negative DWI imaging may receive IV tPA as the DWI imaging information is unable to require before IV tPA. It remains unclear whether DWI-negative stroke would be associated with different clinical prognoses compared with DWI-positive stroke patients. A case-control study with a small sample size (*n* = 43) found better functional outcome at discharge for DWI-positive patients compared with DWI-negative patients [8]. A retrospective study enrolled 89 patients treated with IV tPA within 4.5 hours and found that negative DWI imaging would not increase the risk of symptomatic or asymptomatic cerebral hemorrhage [9]. However, insufficient sample size reduced the validation and generalization of the results from the previous studies [8, 9].

In this study, we aimed to investigate whether negative DWI imaging was related to the early neurological improvement (ENI) or favourable functional outcome at discharge for which group of patients. Considering that only non-contrast CT was available for ischemic stroke patients within 4.5 hours to minimize door-to-needle intervals, MR scans could only be performed after IV tPA.

## Method

### Study design

We conducted a retrospective analysis of the patients that received IV tPA treatment in the neurovascular emergency department (ED) of Tiantan Hospital, Beijing from October 1st, 2018, to October 30th, 2020.

The study was approved by the ethics committee of Beijing Tiantan Hospital (No.: KY2019–019-05). The fully dei-dentified data on the patients enrolled in the current study and its retrospective study design enables this study conducted under a waiver of informed consent by the ethics committee of Beijing Tiantan Hospital. This manuscript is based on the Strengthening the Reporting of Observational Studies in Epidemiology statement to report the results of the current study.

### Participants and data collection

All the patients that received IV tPA treatment were enrolled consecutively. The inclusion criteria were: 1) suspected with acute ischemic stroke in our neurovascular ED, 2) received IV tPA with alteplase (0.9 mg/kg) within 4.5 hours from symptoms onset. The exclusion criteria were: 1) never underwent an MR scan from admission to discharge or had low-quality imaging data such as significant artifacts; 2) had a final diagnosis of ‘stroke mimics’ at discharge; 3) had incomplete or low quality of medical records, especially without NIHSS score at 24 hours or mRS score at discharge.

A neurologist (G.L.) blinded to imaging information extracted the clinical information. The demographic information included age, sex, medical history (hypertension, atrial fibrillation, diabetes mellitus, hyperlipidemia, stroke, and coronary artery disease), alcoholic and tobacco intake, and medication history. Prior antiplatelet/statin therapy referred to taking oral antiplatelet drugs (including aspirin and clopidogrel)/ statin drugs (including lovastatin, simvastatin, pravastatin, fluvastatin, atorvastatin, rosuvastatin, and pivastatin) at the time of index stroke. Door-to-needle time (defined as the time of the hospital arrival to the time of the IV tPA initiation) was also recorded. Disabling stroke [10] was defined as stroke with homonymous hemianopsia, severe limb weakness, severe aphasia, hemineglect, or cortical blindness. Stroke severity was measured by the National Institutes of Health Stroke Scale (NIHSS) score [11].

The cause of ischemic stroke was classified according to the Trial of Org 10,172 in Acute Stroke Treatment (TOAST) classification [12]. Symptomatic intracranial hemorrhage (sICH) events were also recorded based on the national institute of neurological disorders and stroke trial (NINDS) [6] standards. Patients with negative DWI imaging at the first MRI scan and a clear etiology other than ischemic stroke were considered ‘stroke mimics’. A diagnosis of stroke mimics was established by the neurologist (G.L.) that extracted the clinical information with confirmation by two senior neurological professors (Y.X. and X.Z.).

### Imaging assessment

All the MR examinations were performed on a 3.0-T machine (Trio-Tim, Siemens, Erlangen) within 24 hours after IV tPA. The category of DWI-negative or DWI-positive was based on the MR examinations after tPA. The multimodal MR protocol included DWI, fluid-attenuated inversion recovery (FLAIR), and time of flight (TOF) MR angiography (MRA) and was reported before [13]. DWIs were acquired with a single-shot echo-planar imaging with b values of 0 s/mm^2^ and 1000 s/mm^2^, respectively, with the following parameters: repetition time (TR)/echo time (TE) = 3000/75 ms; field of view (FOV) = 23*23 cm; and matrix = 128*128. Apparent diffusion coefficient maps were created from DWI images with b values of 0 s/mm^2^ and 1000 s/mm^2^. FLAIR parameters were: TR/TE/inversion time = 8000/94/2500 ms; FOV = 20*17.6 cm; matrix = 256,179; and flip angle (FA) = 150°. TOF MRA parameters were: TR/TE = 28/3.04 ms; FOV = 20*18 cm; matrix = 256*179; thickness = 0.7 mm; slices per slab = 40; and FA = 13°. The follow-up MR protocol included, but was not limited to, DWI, FLAIR, and TOF MRA [13].

Fazekas scale [14] score was evaluated on the first MR scan after admission. Large vessel occlusion including occlusion of the internal carotid artery, anterior cerebral artery, middle cerebral artery, posterior cerebral artery, and basilar artery confirmed by MRA/CTA. Second imaging (MR or CT scan) after the first MR scan was also evaluated to investigate whether infarction lesion was visible. A neurologist (C.W.) and a neuroradiologist (Y.H.) blinded to clinical information reviewed the imaging data independently. Disagreements were resolved by a third senior neurologist (Y.X.) to reach a consensus.

### Outcome assessment

The primary outcome was defined as NIHSS score reduction≥4 or to baseline within 24 hours from onset (ENI) [6]. The secondary outcome was defined as mRS score 0–1 at discharge (favourable outcome). Modified Rankin Scale (mRS) score was recorded at discharge to evaluate the functional outcome.

### Statistical analysis

Continuous variables were expressed as mean ± SD for normally distributed data and median (interquartile range) for non-normally distributed data. Categorical variables were expressed as number (proportion). Normally distributed data were compared using the student t-test and non-normally distributed data were compared using Mann–Whitney U test. Categorical variables were compared using χ2 test, as appropriate. Variables were tested for collinearity and interactions. All variables with a *P* value < 0.1 in the univariate analysis entered into the multivariable logistic regression models. Skewed continuous variables were transformed into binary variables before entering into multivariable logistic models according to the median. Sensitivity analyses were conducted to exclude patients with mechanical thrombectomy. All statistical analyses were performed with the SPSS 25.0. *P* value< 0.05 was considered statistically significant.

## Results

**Fig. 1:**
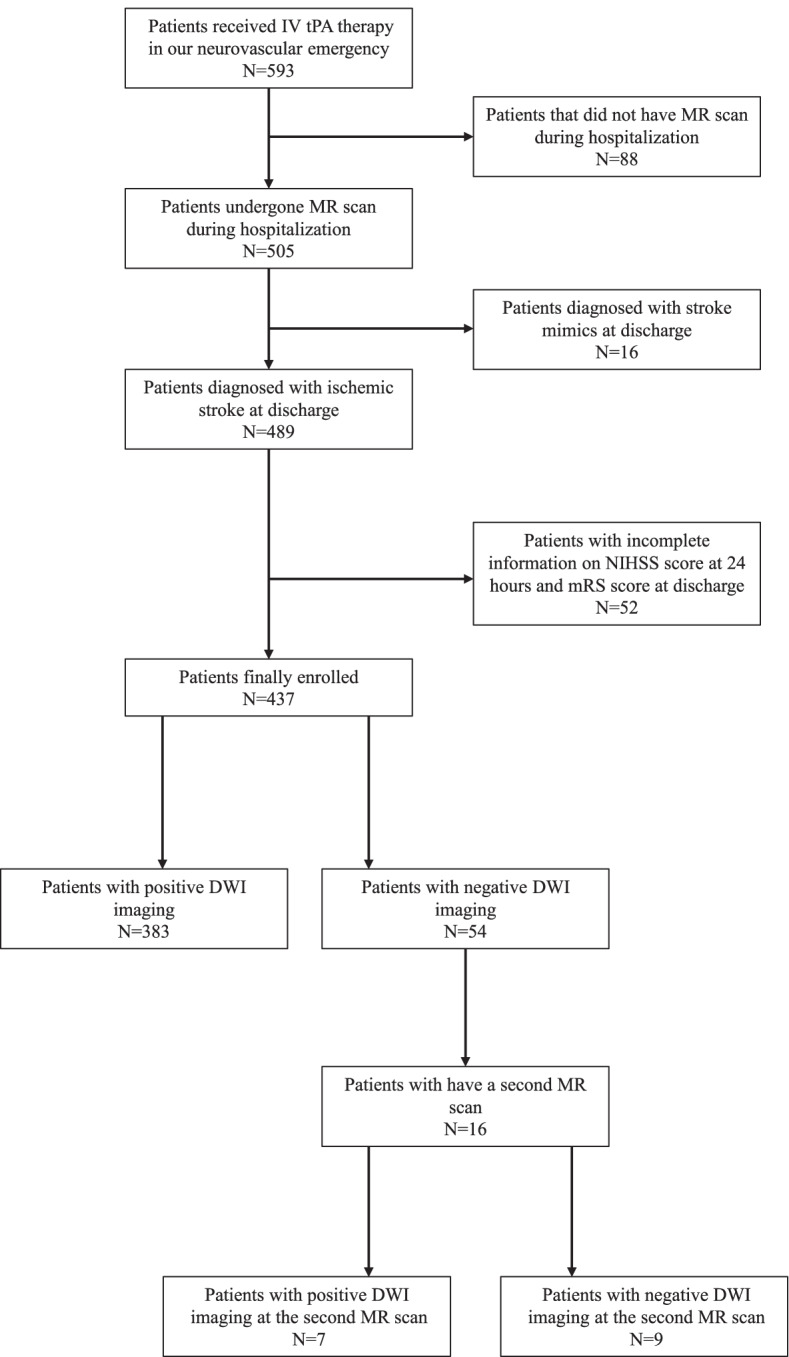
Study flow chart. IV tPA, intravenous thrombolysis tissue plasminogen activator; NIHSS, National Institutes of Health Stroke Scale; DWI, diffusion weighted imaging

From October 1st, 2018 to October 30th, 2020, a total of 593 patients received IV tPA therapy in our neurovascular ED, and 505 of the patients underwent MRI scans during hospitalization. Among the patients that had MRI scans, 3.17% (16/505) of them were diagnosed with stroke mimics at discharge. Another 52 patients were excluded because of incomplete or low quality medical records (Fig. 1).

Overall, 437 patients were included in the final analysis, and the baseline characteristics were summarized in Table 1. The average age was 65.47 ± 11.82 and 73.2% (320/437) of them were male. The median time from symptom onset to IV tPA was 168 (28–215) minutes. The median time from symptom onset to the first MR scan was 26.02 (19.5–27.6) hours. After receiving IV tPA therapy, 39 patients received bridging mechanical thrombectomy therapy. The median admission NIHSS score was 5 (3–9).

**Table 1:** Baseline characteristics between DWI positive and DWI negative group in patients treated with IV tPA

	Overall (*n* = 437)	DWI-negative (*n* = 54)	DWI-positive (*n* = 383)	*P* value
Age, yrs. (mean (SD))	62.5 (11.8)	62.1 (9.9)	62.5 (12.1)	0.80
Male, n (%)	320 (73.2)	37 (68.5)	283 (73.9)	0.40
DNT time, min (median [IQR])	45 [35–61]	41.5 [30.5–55]	45 [35–62]	0.06
Time from symptom onset to IV-tPA, min (median [IQR])	168 [128–215]	160 [116–204]	170 [129.5–215.5]	0.16
Time from symptom onset to the first MR scan, hours (median [IQR])	26.0 [19.5–27.6]	26.9 [25.5–27.7]	25.9 [16.0–27.6]	0.03
Bridging mechanical thrombectomy, n (%)	39 (8.9)	0 (0.0)	39 (10.2)	0.01
Pre-mRS score (%)				0.26
0	329 (75.3)	44 (81.5)	285 (74.4)	
1	57 (13.0)	9 (16.7)	48 (12.5)	
2	26 (5.9)	0 (0.0)	26 (6.8)	
3	15 (3.4)	1 (1.9)	14 (3.7)	
4	9 (2.1)	0 (0.0)	9 (2.3)	
5	1 (0.2)	0 (0.0)	1 (0.3)	
Admission NIHSS score (median [IQR])	5 [3–9]	2.5 [2–5]	5 [3–9]	< 0.001
Admission NIHSS score ≤ 5, n (%)	248 (56.8)	44 (81.5)	204 (53.3)	< 0.001
NIHSS score at 24 hours (median [IQR])	3 [1–6]	1 [0–2]	3 [1–7]	< 0.001
TOAST, n (%)				0.24
LAA	335 (76.7)	45 (83.3)	290 (75.7)	
CE	58 (13.3)	2 (3.7)	56 (14.6)	
SAA	15 (3.4)	3 (5.6)	12 (3.1)	
Other	8 (1.8)	1 (1.9)	7 (1.8)	
Unknown	21 (4.8)	3 (5.6)	18 (4.7)	
Posterior circulation stroke, n (%)	118 (27.0)	21 (38.9)	97 (25.3)	0.04
Disabling stroke, n (%)	390 (89.2)	32 (59.3)	358 (93.5)	< 0.001
Hypertension, n (%)	233 (53.3)	24 (44.4)	209 (54.6)	0.16
Atrial fibrillation, n (%)	42 (9.6)	3 (5.6)	39 (10.2)	0.28
Diabetes mellitus, n (%)	104 (23.8)	12 (22.2)	92 (24.0)	0.77
Hyperlipidemia, n (%)	45 (10.3)	4 (7.4)	41 (10.7)	0.46
Prior stroke, n (%)	87 (19.9)	12 (22.2)	75 (19.6)	0.65
Coronary artery disease, n (%)	61 (14.0)	8 (14.8)	53 (13.8)	0.85
Prior antiplatelet therapy, n (%)	62 (14.2)	8 (14.8)	54 (14.1)	0.89
Prior statin therapy, n (%)	53 (12.1)	8 (14.8)	45 (11.7)	0.52
Smoking, n (%)	228 (52.2)	22 (40.7)	206 (53.8)	0.07
Drinking, n (%)	190 (43.5)	21 (38.9)	169 (44.1)	0.47
Admission SBP level, mmHg (median [IQR])	150 [137–165]	145 [136.5–155.5]	151 [137–166]	0.06
Admission DBP level, mmHg (median [IQR])	88 [80–97]	86 [78.5–91]	89 [80–98]	0.15
Admission serum glucose level, mmol/L (median [IQR])	6.83 [5.9–8.8]	6.41 [5.5–8.0]	6.89 [5.9–8.8]	0.05
HbA1c level, % (median [IQR])	6 [5.7–6.9]	6 [5.7–6.7]	6 [5.7–6.9]	0.92
LDL level, mmol/L (mean (SD))	2.61 (0.9)	2.42 (0.9)	2.63 (0.9)	0.27
Cholesterol level, mmol/L (mean (SD))	4.19 (1.0)	3.87 (1.0)	4.23 (1.0)	0.08
Fazekas scale, (median [IQR])	1 [1–2]	1 [0–1]	1 [1–2]	0.01
Large vessel occlusion, n (%)	99 (22.7)	3 (5.6)	96 (25.1)	0.01
sICH, n (%)	23 (5.3)	1 (1.9)	22 (5.7)	0.38

*DWI* Diffusion weighted imaging, *DNT* Door-to-needle, *IV* Intravenous thrombolysis, *tPA* Tissue plasminogen activator, *mRS* Modified Rankin Scale, *NIHSS* National institutes of health stroke scale, *TOAST* Trial of Org 10,172 in Acute Stroke Treatment, *LAA* Large atherosclerosis artery, *CE* Cardiac embolism, *SAA* Small artery occlusion, *SBP* Systolic blood pressure, *DBP* Diastolic blood pressure, *LDL* Low density lipoprotein, *sICH* Symptomatic intracerebral hemorrhage

### DWI-positive vs. DWI-negative at the first MR scan

At the first MR scan after IV tPA, 12.36% (54/437) patients had negative DWI imaging. In the DWI-negative group, the patients tended to have a longer interval between symptom onset and IV tPA (160 min vs. 170 min, *P* = 0.03), and a lower proportion to undergo bridging mechanical thrombectomy (0 vs. 10.2%, *P* = 0.01). The patients that had negative DWI imaging had milder neurological impairment at admission (median NIHSS score 2.5 vs. 5, *P* < 0.001). The patients that had negative DWI imaging were also found to have a higher proportion of posterior circulation stroke (38.9% vs. 25.3%, *P* = 0.04), a lower proportion of disabling stroke (59.3% vs. 93.5%, *P* < 0.001), lower median Fazekas scale (1[0-1)) vs. 1[1-2]), *P* = 0.01) and lower proportion of large vessel occlusion (5.6% vs. 25.1%, *P* = 0.01) (Table 1).

In the DWI-negative group, 51.9% (28/54) of the patients achieved ENI at 24 hours and 74.1% (40/54) of the patients achieved favourable clinical outcome at discharge. Logistic regression analyses showed that DWI-negative was not related to ENI (crude OR 1.20 [0.30–4.79], *P* = 0.80) or favourable clinical outcome (crude OR 1.92 [0.48–7.75], *P* = 0.36). Adjusted multivariable logistic regression analyses also failed to demonstrate that DWI-negative was related to ENI (adjusted OR 0.93 [0.17–4.91], *P* = 0.93) or favourable clinical outcome (adjusted OR 2.40 [0.48–11.95], *P* = 0.28). Sensitivity analyses were conducted to test the validation of the multivariable logistic regression model. After excluding patients that underwent MT, the multivariable logistic regression model still failed to prove the relationship between DWI-negative and ENI or favourable functional outcome (Table 2). Subgroup analyses were also conducted to between ischemic stroke patients due to anterior or posterior circulation and found that the association between DWI-negative and clinical outcomes were not different between anterior and posterior circulation (Table 3*).*

**Table 2:** Crude and adjusted OR of DWI negative for in-hospital clinical outcomes

	In-hospital clinical outcomes	DWI negative imaging, n (%)	DWI positive imaging, n (%)	Crude OR	95% CI	*P* value	Adjusted^a^ OR	95% CI	*P* value
Multivariable analysis	Early neurological improvement	28 (51.9)	143 (37.3)	1.20	0.30–4.79	0.80	0.93	0.17–4.91	0.93
	Favourable clinical outcome	40 (74.1)	159 (41.5)	1.92	0.48–7.75	0.36	2.40	0.48–11.95	0.28
Sensitivity analysis^b^	Early neurological improvement	28 (51.9)	127 (36.9)	2.01	0.81–4.95	0.13	1.83	0.64–5.22	0.26
	Favourable clinical outcome	40 (74.1)	153 (44.5)	1.73	0.68–4.36	0.25	1.49	0.51–4.34	0.47

*Abbreviations*: *OR* Odd ratio, *CI* Confidence intervals, *DWI* Diffusion weighted imaging

^a^Adjusted for age, male, time from symptom onset to the first MR scan, admission NIHSS score, posterior circulation stroke, smoking, admission SBP level, admission serum glucose level, cholesterol level, Fazekas scale, and large vessel occlusion

^b^Sensitivity analyses after excluding patients treated with bridging mechanical thrombectomy

**Table 3:** Subgroup analyses: DWI negative for in-hospital clinical outcomes

	In-hospital clinical outcomes	Crude OR	95% CI	*P* value
Anterior circulation	Early neurological improvement	0.91	0.14–5.78	0.92
	Favourable clinical outcome	3.45	0.54–22.26	0.19
Posterior circulation	Early neurological improvement	1.86	0.21–16.18	0.58
	Favourable clinical outcome	1.00	0.08–11.93	1.00

*Abbreviations*: *OR* Odd ratio, *CI* Confidence intervals, *DWI* Diffusion weighted imaging

Among the patients that had a negative DWI imaging at the first MR scan after IV tPA, 29.63% (16/54) of the patients had a second MR scan (median interval from symptom onset to the second MR scan 171.2 hours [27.8–234.3]) and 43.75% (7/16) of them had a positive DWI lesion. At the first MR scan, 18.52% (10/54) of the patients had a positive FLAIR lesion correlated to the neurological impairment.

### Favourable functional outcome and ENI

**Fig. 2:**
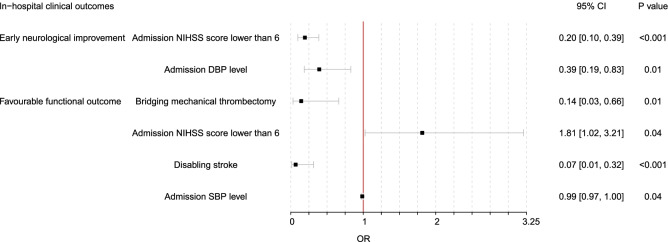
Multivariable logistics regression analysis for the in-hospital clinical outcomes. NIHSS, national institutes of health stroke scale; DBP, diastolic blood pressure; SBP, systolic blood pressure

At 24 hours, 39.1% (171/437) patients had ENI and 45.5% (199/437) patients achieved a favourable functional outcome at discharge. To determine the independent predictors of ENI, DWI-negative, and other variables that showed a significant difference between ENI or not entered into a multivariable logistic regression model (suppl.table 1). In the multivariable logistic regression model, admission NIHSS score ≤ 5 (OR 0.20 [0.10–0.39], *P* < 0.001) and admission diastolic blood pressure (DBP) level (OR 0.39 [0.19–0.83], *P* = 0.01) proved to be independent predictors of ENI (Fig. 2). To determine predictors of favourable functional outcome, another multivariable logistic regression model was conducted after including DWI-negative and other variables that showed a significant difference between favourable functional outcome or not at discharge (suppl.table 2). Bridging mechanical thrombectomy (MT) (OR 0.14 [0.03–0.66], *P* = 0.01), admission NIHSS score ≤ 5 (OR 1.81 [1.02–3.21], *P* = 0.04), disabling stroke (OR 0.07 [0.01–0.32], *P* < 0.001), and admission SBP level (OR 0.99 [0.97–1.00], *P* = 0.04), proved to be independent predictors of favourable functional outcome at discharge (Fig. 2).

## Discussion

The current showed that 12.36% of the ischemic stroke patients had negative DWI imaging at the first MR scan after IV tPA therapy. However, DWI-negative was not an independent predictor of ENI or favourable functional outcome at discharge for ischemic stroke treated with intravenous thrombolysis. Our study had a larger sample size relative to previous studies [8, 9] (sample size< 100) that enhanced the validation and generalization of the results of our study.

In accordance with previous studies [4, 8, 9, 15, 16], this study showed that 12.36% of the patients that received IV tPA within 4.5 hours had negative DWI imaging. However, the rate of negative DWI imaging was lower than that of another study [17] (29%) with lower admission NIHSS score, enrollment of TIA, and different imaging parameters discussed later.

Several factors were reported to be related to DWI-negative stroke including lower admission NIHSS score [8, 9, 18, 19], and involvement of posterior circulation [8] which were also shown in our study. However, our study found that patients had longer intervals from stroke onset to MR scan in the DWI-negative group. A potential explanation was the lower admission NIHSS score in our study. A prospective observational study [19] enrolled patients with minor stroke (median NIHSS score = 2) and found patients with DWI-negative tended to have a prolonged interval from stroke onset to MR scan compared with patients with DWI-positive due to rapid cellular edema normalization with smaller infarcts in minor stroke.

Apart from clinical factors, imaging parameters could also be related to negative DWI imaging. Modification of parameters with advanced imaging protocol could be beneficial to increase the rate of positivity of DWI imaging including reducing the slice thickness, increasing the b-value, adding coronal or sagittal DWI imaging, and increasing the number of diffusion directions [4, 20]. Minor strokes and strokes in the brainstem tended to have smaller infarcts that could be missed in DWI imaging with large slice thickness [20]. A retrospective, observational study utilized 2 mm thin-section DWI imaging and reported a 13.2% of the included patients (*n* = 5271) had negative DWI imaging at the initial MR scan [18], lower than the proportion (29%) reported in a forehead study that utilized 5 mm thin-section DWI imaging [17].

DWI imaging is sensitive to the restriction of water diffusion, also known as cytotoxic edema, one of the most usual tissue changes when the ischemic stroke occurred. However, stroke may also be due to milder ischemic tissue changes including cerebral oligaemia or cerebral blood flow reduction tended to be invisible on DWI imaging. Cerebral oligaemia or cerebral blood flow reduction was not as severe as cytotoxic edema and manifested as minor ischemic symptoms and lower NIHSS score [8, 21]. Perfusion imaging could help to quantify the severity of the ischemic lesion. When the cerebral blood flow (CBF) dropped to the level of 20–25 ml/100 g/min, an ischemic lesion could be detected on DWI imaging, thus an ischemic lesion is invisible on DWI imaging if the CBF value was between 25 and 35 ml/100 g/min while ischemic symptoms could be onset [17]. However, these milder ischemic tissue changes are unlikely to exclude large vessel occlusion and subsequent neurological deterioration, implying reperfusion therapy is still rational to achieve recanalization and improve functional outcomes. In our study, 5.6% of the DWI-negative patients showed large vessel occlusion on MRA/CTA. However, IV tPA therapy could increase the risk of hemorrhagic event, especially sICH, which was the most harmful adverse events due to IV tPA and worsen the clinical outcomes. Our study and previous studies [8, 9] showed a similar risk of sICH between DWI-positive and DWI-negative patients receiving IV tPA therapy.

In our study, about 30% of the patients had a second MR scan and less than half of the patients who had a second MR scan had positive DWI imaging at the follow-up MR scan. Another observational study that enrolled TIA and ischemic stroke reported a rate of 22.5% that had positive DWI imaging at the follow-up MR scan while the first DWI imaging was negative [18]. When the ischemic infarction occurred initially, the biological changes were not severe enough to be detected via DWI imaging. As the ischemic event persisted or worsen, especially cytotoxic edema occurred, the ischemic lesion became visible on the DWI scan. Each hour delay of DWI scan from ischemic symptom onset increased the chances of positivity of DWI by 4% [21]. Hence, a follow-up MR scan may be necessary after negative DWI imaging at the first MR scan to confirm the diagnosis of ischemic stroke.

### Limitations

First, this study was based on a retrospective study design in a single stroke center. However, the neurovascular ED-based in Beijing Tiantan hospital received over 1000 patients suspected of acute ischemic stroke and had over 300 patients who received IV tPA each year, accumulating large sets of data on IV tPA. Second, there might be observational bias as the MR imaging was read manually. However, two separate readers with different medical backgrounds reviewed all the MR imaging independently blinded to clinical information to minimize the bias. Third, the functional outcome at long-term follow-up was not available in our study. Considering the pandemic of COVID-19 and population mobility of Beijing, the capital of China, face-to-face follow-up is unlikely to achieve. Telephone interviews were not able to avoid bias. Hence, in our study, we canceled analysis on long-term outcomes. Fourth, we did not conduct perfusion imaging in the included patients of our study as perfusion imaging was not conducted routinely in ischemic stroke patients considering its time-consuming and side effects of contrast agents. However, perfusion imaging might provide additional imaging information and future studies were suggested to perform perfusion imaging in DWI-negative patients.

## Conclusion

DWI-negative is not associated with ENI or favourable functional outcome at discharge for acute ischemic stroke patients treated with intravenous thrombolysis.

## Supplementary Information

**Additional file 1: Supple. Table 1.** Baseline characteristics between ENI and no ENI group in patients treated with IV tPA.

**Additional file 2: Supple. Table 2.** Baseline characteristics between mRS≥2 and mRS 0-1 group in patients treated with IV tPA.

## Data Availability

The datasets used and/or analysed during the current study available from the corresponding author on reasonable request.
