# Supplementary material for: In-hospital clinical outcomes in diffusion weighted imaging-negative stroke treated with intravenous thrombolysis
Source: BMC Neurol. 2022 Sep 15;22:349. doi: 10.1186/s12883-022-02878-w (PMC9476428; doi:10.1186/s12883-022-02878-w)
Supplement: Supplementary file 1 — Additional file 1: Supple. Table 1. Baseline characteristics between ENI and no ENI group in patients treated with IV tPA. [file 12883_2022_2878_MOESM1_ESM.docx]

Supple. Table 1. Baseline characteristics between ENI and no ENI group in patients treated with IV tPA

|  | Overall  (n=437) | No ENI  (n=266) | ENI  (n=171) | P value |
| --- | --- | --- | --- | --- |
| Age, yrs (mean (SD)) | 62.5 (11.8) | 62.8 (11.6) | 61.9 (12.2) | 0.44 |
| Male, n (%) | 320 (73.2) | 200 (75.2) | 120 (70.2) | 0.25 |
| DNT time (median [IQR]) | 45 [35-61] | 43 [34.3-59] | 47 [35-64.5] | 0.20 |
| Time from symptom onset to IV-tPA, min (median [IQR]) | 168 [128-215] | 170.5 [130-219.8] | 165 [125-207] | 0.16 |
| Time from symptom onset to the first MR scan, hours (median [IQR]) | 26.0 [19.5-27.6] | 26.0 [20.7-27.4] | 26.0 [13.2-28.1] | 0.57 |
| Bridging mechanical  thrombectomy, n (%) | 39 (8.9) | 23 (8.6) | 16 (9.4) | 0.80 |
| Pre-mRS score (%) |  |  |  | 0.33 |
| 0 | 329 (75.3) | 191 (71.8) | 138 (80.7) |  |
| 1 | 57 (13.0) | 37 (13.9) | 20 (11.7) |  |
| 2 | 26 (5.9) | 19 (7.1) | 7 (4.1) |  |
| 3 | 15 (3.4) | 11 (4.1) | 4 (2.3) |  |
| 4 | 9 (2.1) | 7 (2.6) | 2 (1.2) |  |
| 5 | 1 (0.2) | 1 (0.4) | 0 (0.0) |  |
| Admission NIHSS score (median [IQR]) | 5 [3-9] | 4 [3-7] | 7 [3-11] | <0.001 |
| Admission NIHSS score≤5 (%) | 248 (56.8) | 178 (66.9) | 70 (40.9) | <0.001 |
| NIHSS score at 24 hours (median [IQR]) | 3 [1-6] | 4 [2-8] | 1 [0-3] | <0.001 |
| TOAST, n (%) |  |  |  | 0.52 |
| LAA | 335 (76.7) | 206 (77.4) | 129 (75.4) |  |
| CE | 58 (13.3) | 33 (12.4) | 25 (14.6) |  |
| SAA | 15 (3.4) | 11 (4.1) | 4 (2.3) |  |
| Other | 8 (1.8) | 3 (1.1) | 5 (2.9) |  |
| Unknown | 21 (4.8) | 13 (4.9) | 8 (4.7) |  |
| Hypertension, n (%) | 233 (53.3) | 151 (56.8) | 82 (48.0) | 0.07 |
| Atrial fibrillation, n (%) | 42 (9.6) | 26 (9.8) | 16 (9.4) | 0.89 |
| Diabetes mellitus, n (%) | 104 (23.8) | 66 (24.8) | 38 (22.2) | 0.54 |
| Hyperlipidemia, n (%) | 45 (10.3) | 22 (8.3) | 23 (13.5) | 0.08 |
| Prior stroke, n (%) | 87 (19.9) | 53 (19.9) | 34 (19.9) | 0.99 |
| Coronary artery disease, n (%) | 61 (14.0) | 34 (12.8) | 27 (15.8) | 0.38 |
| Prior antiplatelet therapy, n (%) | 62 (14.2) | 31 (11.7) | 31 (18.1) | 0.06 |
| Prior statin therapy, n (%) | 53 (12.1) | 21 (7.9) | 32 (18.7) | 0.01 |
| Smoking, n (%) | 228 (52.2) | 148 (55.6) | 80 (46.8) | 0.07 |
| Drinking, n (%) | 190 (43.5) | 126 (47.4) | 64 (37.4) | 0.04 |
| Admission SBP level, mmHg (median [IQR]) | 150 [137-165] | 154 [140-167] | 146 [135-161.5] | 0.04 |
| Admission DBP level, mmHg (median [IQR]) | 88 [80-97] | 90 [80-98] | 86 [77.5-95] | 0.02 |
| Admission serum glucose level, mmol/L (median [IQR]) | 6.8 [5.9-8.8] | 6.8 [5.9-9.0] | 6.8 [5.9-8.4] | 0.60 |
| HbA1c level, % (median [IQR]) | 6.0 [5.7-6.9] | 6.0 [5.7-6.8] | 6.0 [5.7-7.0] | 0.57 |
| LDL level, mmol/L (mean (SD)) | 2.6 (0.9) | 2.7 (0.9) | 2.5 (0.9) | 0.03 |
| Cholesterol level, mmol/L (mean (SD)) | 4.2 (1.0) | 4.3 (0.9) | 4.0 (1.1) | 0.02 |
| DWI positive at the first MR scan, n (%) | 383 (87.6) | 240 (90.2) | 143 (83.6) | 0.04 |
| Fazekas scale, (median [IQR]) | 1 [1-2] | 1 [1-2] | 1 [1-1] | 0.19 |
| Large vessel occlusion, n (%) | 99 (22.7) | 58 (21.8) | 41 (24.0) | 0.60 |
| sICH, n (%) | 23 (5.3) | 16 (6.0) | 7 (4.1) | 0.38 |

ENI, early neurological improvement; DWI, diffusion weighted imaging; DNT, door-to-needle; IVT, intravenous thrombolysis; tPA, tissue plasminogen activator; mRS, modified Rankin Scale; NIHSS, national institutes of health stroke scale; TOAST, Trial of Org 10172 in Acute Stroke Treatment; LAA, large atherosclerosis artery; CE, cardiac embolism; SAA, small artery occlusion; SBP, systolic blood pressure; DBP, diastolic blood pressure; LDL, low density lipoprotein; sICH, symptomatic intracerebral hemorrhage
