# Supplementary material for: In-hospital clinical outcomes in diffusion weighted imaging-negative stroke treated with intravenous thrombolysis
Source: BMC Neurol. 2022 Sep 15;22:349. doi: 10.1186/s12883-022-02878-w (PMC9476428; doi:10.1186/s12883-022-02878-w)
Supplement: Supplementary file 2 — Additional file 2: Supple. Table 2. Baseline characteristics between mRS≥2 and mRS 0-1 group in patients treated with IV tPA. [file 12883_2022_2878_MOESM2_ESM.docx]

Supple. Table 2. Baseline characteristics between mRS≥2 and mRS 0-1 group in patients treated with IV tPA

|  | Overall  (n=437) | Discharge mRS≥2  (n=238) | Discharge mRS 0-1  (n=199) | P value |
| --- | --- | --- | --- | --- |
| Age, yrs (mean (SD)) | 62.5 (11.8) | 63.0 (11.7) | 61.9 (12.0) | 0.35 |
| Male, n (%) | 320 (73.2) | 170 (71.4) | 150 (75.4) | 0.35 |
| DNT time (median [IQR]) | 45 [35-61] | 45 [36-61] | 43 [33-62] | 0.28 |
| Time from symptom onset to IV-tPA, min (median [IQR]) | 168 [128-215] | 175 [135-222] | 165 [120-204.5] | 0.04 |
| Time from symptom onset to the first MR scan, hours (median [IQR]) | 26.0 [19.5-27.6] | 25.6 [6.6-27.5] | 26.5 [24.9-27.8] | 0.01 |
| Bridging mechanical  thrombectomy, n (%) | 39 (8.9) | 33 (13.9) | 6 (3.0) | <0.001 |
| Pre-mRS score (%) |  |  |  | 0.17 |
| 0 | 329 (75.3) | 170 (71.4) | 159 (79.9) |  |
| 1 | 57 (13.0) | 34 (14.3) | 23 (11.6) |  |
| 2 | 26 (5.9) | 15 (6.3) | 11 (5.5) |  |
| 3 | 15 (3.4) | 10 (4.2) | 5 (2.5) |  |
| 4 | 9 (2.1) | 8 (3.4) | 1 (0.5) |  |
| 5 | 1 (0.2) | 1 (0.4) | 0 (0.0) |  |
| Admission NIHSS score (median [IQR]) | 5 [3-9] | 7 [4-11] | 3 [2-5.5] | <0.001 |
| Admission NIHSS score≤5 (%) | 248 (56.8) | 101 (42.4) | 147 (73.9) | <0.001 |
| NIHSS score at 24 hours (median [IQR]) | 3 [1-6] | 5 [3-10] | 1 [0-2] | <0.001 |
| TOAST, n (%) |  |  |  | 0.21 |
| LAA | 335 (76.7) | 177 (74.4) | 158 (79.4) |  |
| CE | 58 (13.3) | 38 (16.0) | 20 (10.1) |  |
| SAA | 15 (3.4) | 6 (2.5) | 9 (4.5) |  |
| Other | 8 (1.8) | 6 (2.5) | 2 (1.0) |  |
| Unknown | 21 (4.8) | 11 (4.6) | 10 (5.0) |  |
| Hypertension, n (%) | 233 (53.3) | 136 (57.1) | 97 (48.7) | 0.08 |
| Atrial fibrillation, n (%) | 42 (9.6) | 27 (11.3) | 15 (7.5) | 0.18 |
| Diabetes mellitus, n (%) | 104 (23.8) | 58 (24.4) | 46 (23.1) | 0.76 |
| Hyperlipidemia, n (%) | 45 (10.3) | 21 (8.8) | 24 (12.1) | 0.27 |
| Prior stroke, n (%) | 87 (19.9) | 45 (18.9) | 42 (21.1) | 0.57 |
| Coronary artery disease, n (%) | 61 (14.0) | 34 (14.3) | 27 (13.6) | 0.83 |
| Prior antiplatelet therapy, n (%) | 62 (14.2) | 33 (13.9) | 29 (14.6) | 0.83 |
| Prior statin therapy, n (%) | 53 (12.1) | 24 (10.1) | 29 (14.6) | 0.15 |
| Smoking, n (%) | 228 (52.2) | 123 (51.7) | 105 (52.8) | 0.82 |
| Drinking, n (%) | 190 (43.5) | 99 (41.6) | 91 (45.7) | 0.39 |
| Admission SBP level, mmHg (median [IQR]) | 150 [137-165] | 154 [139-168] | 147.5 [137-162] | 0.06 |
| Admission DBP level, mmHg (median [IQR]) | 88 [80-97] | 90 [80-98] | 87 [80-96] | 0.26 |
| Admission serum glucose level, mmol/L (median [IQR]) | 6.8 [5.9-9.0] | 7.3 [5.9-9.3] | 6.4 [5.9-8.3] | 0.02 |
| HbA1c level, % (median [IQR]) | 6.0 [5.7-6.9] | 6.1 [5.8-7.0] | 5.9 [5.6-6.6] | 0.08 |
| LDL level, mmol/L (mean (SD)) | 2.6 (0.9) | 2.6 (0.9) | 2.6 (0.9) | 0.59 |
| Cholesterol level, mmol/L (mean (SD)) | 4.2 (1.0) | 4.2 (1.0) | 4.2 (1.0) | 1 |
| DWI positive at the first MR scan, n (%) | 383 (87.6) | 224 (94.1) | 159 (79.9) | <0.001 |
| Fazekas scale, (median [IQR]) | 1 [1-2] | 1 [1-2] | 1 [1-1] | 0.01 |
| Large vessel occlusion, n (%) | 99 (22.7) | 72 (30.3) | 27 (13.6) | <0.001 |
| sICH, n (%) | 23 (5.3) | 20 (8.4) | 3 (1.5) | 0.01 |

DWI, diffusion weighted imaging; DNT, door-to-needle; IVT, intravenous thrombolysis; tPA, tissue plasminogen activator; mRS, modified Rankin Scale; NIHSS, national institutes of health stroke scale; TOAST, Trial of Org 10172 in Acute Stroke Treatment; LAA, large atherosclerosis artery; CE, cardiac embolism; SAA, small artery occlusion; SBP, systolic blood pressure; DBP, diastolic blood pressure; LDL, low density lipoprotein; sICH, symptomatic intracerebral hemorrhage
